# 

**Published:** 1884-04

**Authors:** 


### EDITORIAL.

### The Southern Dental Association.

The sixteenth annual meeting of this body will be held in Lexington, Ky., on Tuesday, May 6th, 1884.

The Executive Committee have made arrangements with all the railroads and hotels for reduced rates for travel and entertainment. The meeting promises to be one of unusual interest, both professionally and socially.

The following subjects are on the programme for consideration:

1. Dental Education.
2. Dental Hygiene.
3. Pathology and Therapeutics.
4. Histology and Microscopy.
5. Chemistry.
6. Operative Dentistry.
7. Mechanical Dentistry.
8. Dental Literature.

With such a list of subjects there will be ample opportunity for every one to take part.

DR. N. J. MCKELLOPS, President, St. Louis, Mo.
DR. A. O. RAWLS, Chairman Ex. Com., Lexington, Ky.

---

THE Mad River Valley Dental Society will hold its annual meeting in the parlor of the Phillips House, Dayton, O., May 20, 1884. Sessions at 10 o'clock A. M. and 2 and 7 P. M.

SUBJECTS FOR DISCUSSION.

1. The Value of Pulpless Teeth and Roots.
2. Inflammation.
3. Alveolar Abscess.
4. The Promotion of Osseous Development.
5. Facial Neuralgia.

It is hoped that every member, and every dentist who can, will be present.

---

### Meeting.

The annual meeting of the Dental Society of the State of New York will be held at Albany, Wednesday and Thursday, May 14th and 15th.

The Board of Censors will meet and examine candidates for the degree of M.D.S., at the same place, Tuesday, May 13th. No examinations will be held after Tuesday evening.

For information as to requirements of the Board, address,

DR. FRANK FRENCH, Rochester, N. Y.

J. EDWARD LINN, Secretary.

### CONTENTS.

COMMUNICATIONS.

Phosphate of Lime and the Teeth.... 163
Carbolic Acid.... 173

PROCEEDINGS.

Fortieth Annual Meeting Miss. Valley Dental Association.... 184

SELECTIONS.

Salmagundi.... 208

PROPOSITIONS.

A Proposition.... 212

EDITORIAL.

The S uthern Dental Association.... 213

Mad River Dental Society.... 214

Dental Society State of New York.... 214

### THE DENTAL REGISTER,

A MONTHLY MAGAZINE DEVOTED TO THE INTERESTS OF THE

**DENTAL PROFESSION.**

*Terms Reduced to* $2.00 *Per Year. Single Copies,* 25 *Cents.*

**EDITED BY PROF. J. TAFT, D.D.S.**

AND

**Published by SPENCER & CROCKER, Ohio Dental and Surgical Depot.**

The volume commences in January and closes in December.

The REGISTER being issued monthly is always fresh, therefore Indispensable to the practitioner who desires to keep posted up with the new inventions and improvements which are daily developed. Neither expense nor effort will be spared to give value and variety to its contents. Articles will be illustrated with well executed engravings whenever they will add to the interest or assist to explanation.

Its extensive circulation and frequency of issue make it one of the BEST DENTAL ADVERTISING MEDIUMS in the United States.

All subscriptions must begin with January or July.

Local or special notices, five lines or less, will be inserted for $3 each insertion; over five lines, 50 cts. per line.

All advertising bills payable in advance, or at furthest, after the issue of the first number containing the advertisement. All transient advertisements to be paid for in advance.

☞CONTRIBUTIONS SOLICITED.

Exclusively Editorial Communications should be addressed to the Editor.

The latest number of the REGISTER may be ordered with or without other goods at any time, and all letters should be addressed to

**SPENCER & CROCKER, Ohio Dental & Surgical Depot, Cincinnati, O.**

